# Ranking factors across multiple domains in predicting adolescent mental health: a Bayesian machine learning approach

**DOI:** 10.1186/s13034-025-00969-3

**Published:** 2025-10-14

**Authors:** Shan Zhao, Xuanjing Li, Xiang Gao, Yipeng Lv, Yang Cao, Gaofeng Mi, Hui Wang, Li Niu, Yan Li

**Affiliations:** 1 School of Public Health, Shanghai Jiao Tong University School of Medicine, Shanghai, China; 2 Qu Xian High School, Dazhou, Sichuan China; 3 Qu Xian Second Middle School, Dazhou, Sichuan China; 4 Faculty of Psychology, Beijing Normal University, Beijing, China; 5 Department of Pediatrics, Icahn School of Medicine at Mount Sinai, New York, NY USA; 6 Department of Population Health Science and Policy, Icahn School of Medicine at Mount Sinai, New York, NY USA

**Keywords:** Mental health, Depression, Anxiety, Sleep quality, Adolescents, Machine learning

## Abstract

**Background:**

The prevalence of mental health problems among adolescents is on the rise globally, and is a pressing public health concern in many developing countries, including China. While a growing body of epidemiological research has identified potential factors affecting adolescent mental health, few have considered both risk and protective factors across multiple domains or utilized machine learning approaches to identify and rank these factors.

**Methods:**

This is a cross-sectional study based on data from 3,526 adolescent participants aged 11–15 years in the Qu County Study in China, and aims to identify and rank factors across five domains—including sociodemographic factors, academic functioning, extracurricular activities, life experiences, and resilience factors—in predicting adolescent mental health outcomes. A Bayesian machine learning approach is used to identify and rank important factors in predicting adolescent mental health outcomes, including depressive symptoms, anxiety symptoms, and sleep quality.

**Results:**

The machine learning models showed satisfactory predictive performance across outcomes (pseudo-R² = 0.24–0.61; RMSE = 0.65–3.60). Experiences of life stress, benevolent events, environmental sensitivity, and shift-and-persist coping strategies were common top predictors in predicting depressive symptoms, anxiety symptoms, and sleep quality. Stress mindset and expressive suppression strategies were unique predictors of sleep quality and depressive symptoms, respectively.

**Conclusions:**

Our results revealed the importance of life experience and resilience factors in predicting adolescent mental health. Future studies should investigate the causal relationship between these understudied factors and adolescent mental health.

**Supplementary Information:**

The online version contains supplementary material available at 10.1186/s13034-025-00969-3.

## Introduction

The World Health Organization (WHO) defines mental health as a state of well-being in which individuals can cope with the normal stressors of life and function productively [1]. Mental health problems are the leading cause of morbidity and mortality among adolescents globally [2]. Without proper interventions, mental health problems during adolescence may have lasting impact on physical health, social life, and longevity in later years [3]. Following the COVID-19 pandemic, several meta-analysis studies found that 1 in 4 children and adolescents under age 18 reported having symptoms of depression or sleep disturbances, and 1 in 5 reported experiencing anxiety symptoms [4, 5]. These high rates of mental health problems almost doubled those reported prior to the pandemic [4, 5]. A recent nationwide survey in China has also demonstrated comparable prevalence rates for mental health problems among Chinese adolescents (i.e., 23.3% for depression and 33.0% for poor sleep quality) [6]. Given the notable rise in the prevalence of adolescent mental health problems [7, 8], there is an urgent need for an improved understanding of risk and protective factors of adolescent mental health to inform effective and actionable prevention strategies. In this context, the present study focuses on depression, anxiety, and poor sleep, which represent three of the most common, burdensome, and interrelated mental health concerns in adolescence [5, 9].

Bronfenbrenner’s ecological systems theory [10] provides a useful framework for understanding how factors across multiple levels—including family environment, individual psychosocial characteristics, and behavioral patterns—influence adolescent development. Guided by this theory, prior research has identified a range of risk and protective factors for adolescent mental health [11–16]. For example, adverse family environments such as low socioeconomic status and exposure to domestic violence (macrosystem and microsystem) have been linked to poorer adolescent mental health [17, 18]. In contrast, benevolent experiences and individual resilience (microsystem and individual factors) function as important protective factors [19–22]. Resilience, in this context, refers to the capacity to adapt effectively to adversity or significant stressors and to recover while maintaining functioning [23]. Based on the existing literature, the present study organizes predictors into five domains—sociodemographic characteristics, academic functioning, extracurricular activities, life experiences, and resilience. These domains align with different ecological layers and have been consistently emphasized in both ecological theory and empirical research as critical to adolescent development and mental health.

Although prior research has improved our understanding of factors associated with adolescent mental health, most studies have relied on traditional regression-based analyses. These approaches assume linearity, normality, and independence, assumptions that often do not hold in real-world data [24]. Moreover, regression models typically explain only a modest proportion of the variance in mental health outcomes and are often underpowered to simultaneously examine a comprehensive set of theory-driven factors across multiple domains. To address these limitations, recent studies have begun to apply machine learning techniques, which can accommodate large, complex datasets and generate insights under more flexible assumptions [25, 26].

Despite these advances, the evidence base remains limited in the following ways. First, much of the research has focused on predictors of single mental health outcomes, overlooking transdiagnostic factors that may influence multiple outcomes simultaneously. This is important because some mental health outcomes, such as sleep disturbances and depressive symptoms, are closely correlated and often predict one another [27, 28]. A deeper understanding of the shared predictors could therefore help identify adolescents at heightened vulnerability. Second, previous research has mostly concentrated on risk factors of adolescent mental health [29], with comparatively little attention to protective factors. For instance, structural risk factors such as family financial strain are consistently linked to adolescent depression [30]; yet these risks can be difficult to modify, particularly in resource-limited contexts. By contrast, protective factors such as coping strategies and resilience may represent more feasible and scalable targets for prevention. Third, although machine learning has been increasingly adopted, approaches with stronger predictive performance and more rigorous methods for quantifying predictor importance are still needed. Bayesian Additive Regression Trees (BART), a Bayesian ensemble method, offers superior predictive performance and a principled framework for evaluating predictor importance [31, 32]. BART has been applied successfully to identify and rank key predictors of chronic conditions such as coronary heart disease, stroke, and cancer [33–35]. Extending this approach to adolescent mental health holds promise for identifying both risk and protective factors across multiple mental health outcomes with greater robustness.

Addressing these gaps, the present study applies the BART approach to identify and rank factors across five domains—including sociodemographic factors, academic functioning, extracurricular activities, life experiences, and resilience factors—in relation to adolescent mental health. We also examine the associations between these predictors and mental health outcomes. We hypothesize that predictors from all five domains will contribute to these outcomes (i.e., depression, anxiety, and sleep quality), with some emerging as transdiagnostic factors and others as outcome-specific. We then apply principled variable selection algorithms informed by the machine learning outputs to evaluate their relative contributions. Collectively, these analyses aim to advance understanding of multilevel risk and protective factors for adolescent mental health.

## Methods

### Participants

The data for the current study came from the Qu County Study, an ongoing cohort study on the physical and mental health of adolescents in China. We used baseline data collected in October 2023 through paper-and-pencil surveys administered at the two largest public schools in Qu County, Sichuan Province, China. All seventh- and eighth-grade students were invited to participate. A total of 4,983 students were eligible, and 4,897 agreed to participate, yielding a 98.3% participation rate at recruitment. Surveys were administered during regular school hours, with adolescents completing the questionnaires in their respective classrooms under the supervision of trained research assistants. The research assistants explained the study purpose and procedures, obtained informed consent, and addressed any questions from the adolescents.

Of the 4897 adolescents who provided informed consent and completed the questionnaires, 103 were excluded due to missing demographic or outcome data. An additional 1268 were excluded due to missing data on key predictor variables (*n* = 206 for missing environmental factors, *n* = 399 for missing academic functioning variables, *n* = 116 for missing lifestyle information, and *n* = 547 for missing psychological characteristics). Our final analytical sample included 3,526 adolescents, aged 11 to 15 years, with a mean age of 13.09 years (*SD* = 0.72), representing all complete cases required for analysis. This sample size is in line with prior applications of BART and is appropriate for stable estimation and mitigating overfitting [36]. Among them, 47.1% were male. Younger participants and female students were more likely to be excluded due to missing data compared to those in the final sample (*p*s < 0.001). Table 1 presents an overview of the sociodemographic and mental health characteristics of the sample.

**Table 1:** Sample characteristics

Domain	Variable	M (SD) / *n* (%)	Min–Max
Sociodemographic characteristics	Sex		–
	Male	1659 (47.1%)	
	Female	1867 (52.9%)	
	Age	13.09 (0.72)	11–15
	Parental highest educational level		–
	Middle school or below	1530 (43.4%)	
	High school	898 (25.5%)	
	2- or 3-year college	319 (9%)	
	4-year college or bachelor’s degree or above	779 (22.1%)	
	Subjective SES^a^	4.85 (1.27)	1–10
Extracurricular activities	Physical activity (h/week)	2.88 (3.82)	0–65
	Reading time (h/week)	3.81 (4.99)	0–60
	Internet use (h/week)	5.16 (6.72)	0–60
	Art (h/week)	1.30 (2.38)	0–56
Academic functioning	Mindset of intelligence^b^	4.62 (0.99)	1–6
	Educational aspiration		–
	High school or below	170 (4.8%)	
	2- or 3-year college	115 (3.3%)	
	4-year college or bachelor’s degree	1624 (46%)	
	master’s degree or above	1617 (45.9%)	
	Learning goals		
	Performance-oriented learning goal	3.90 (1.15)	1–6
	Mastery-oriented learning goal	4.16 (1.10)	1–6
	School engagement	3.20 (0.36)	1.27-4
Life experience	Experience of life stressors	2.07 (0.48)	1–4
	Experience of benevolent events	7.42 (2.05)	0–10
Resilience	Future orientation	3.10 (0.57)	1–4
	Meaning in life	4.73 (1.39)	1–7
	Gender norm attitude^c^	2.81 (0.74)	1–5
	Perceived economic inequality	2.39 (0.90)	1–5
	Environmental sensitivity	5.08 (0.76)	1–7
	Shift-and-persist strategies	2.86 (0.51)	1–4
	Emotion regulation strategies		
	Expressive suppression	4.25 (1.15)	1–7
	Cognitive reappraisal	4.51 (0.95)	1–7
	Stress mindset^d^	1.88 (0.60)	0–4
Mental health outcomes	Depressive symptoms	9.99 (5.70)	0–30
	Anxiety symptoms	6.16 (4.92)	0–21
	Poor sleep quality	2.25 (0.75)	1–4

^a^ Individual perceived social status compared with others in their living city

^b^ Individual beliefs about whether intelligence is malleable or not

^c^ Individual stereotypical attitudes toward masculinities and femininities

^d^ Individual beliefs about whether stress is enhancing or debilitating

### Measures

#### Outcome variables

We examined three outcome variables in this study: depressive symptoms, anxiety symptoms, and sleep quality. Depressive symptoms were assessed using the 10-item version of the Center for Epidemiologic Studies Depression Scale (CESD) [37]. The CESD is a well-validated measure with strong reliability and validity demonstrated across diverse populations, including Chinese adolescents [38]. Participants were asked to rate the frequency of each of the 10 symptoms that were provided (e.g., felt depressed). Respondents were given on a 4-point Likert scale from 0 (*rarely or none of the time*) to 3 (*all of the time*) and were summed to create a total score ranging from 0 to 30. A higher score therefore indicates more depressive symptoms. A cutoff score of 10 has been suggested in previous research [39]; thus, participants with scores above this threshold were considered to have clinically significant depressive symptoms. In this study, the Cronbach’s coefficient α was 0.84 and the McDonald’s omega reliability coefficient was 0.85.

Anxiety symptoms were measured using the Generalized Anxiety Disorder Scale (GAD-7) [40, 41], a well-validated and widely used instrument for adolescents, which has demonstrated strong psychometric properties in Chinese populations. Participants were asked to evaluate the frequency of each of the seven listed symptoms on a 4-point Likert scale (0 = *rarely or none of the time* to 3 *= all of the time*). The scale ranges from 0 to 21, and anxiety symptoms can be indicated as mild, moderate, or severe, as indicated by scores of ≥ 5, ≥10, and ≥ 15, respectively [41]. The scale demonstrated excellent internal consistency, with Cronbach’s α = 0.90 and McDonald’s ω = 0.90.

Sleep quality was assessed using the single subjective sleep quality item from the Pittsburgh Sleep Quality Index, which has been shown to be reliable and valid when compared with polysomnography [42]. Participants were asked to rate their overall sleep quality during the last month, with responses ranging from 1 (*excellent*) to 4 (*poor*), with a higher score representing poorer sleep quality. The single-item sleep quality measure is widely used in large-scale studies and has demonstrated acceptable test–retest reliability and construct validity across diverse samples, including Chinese adolescents [43, 44].

### Predictors

Consistent with Bronfenbrenner’s ecological systems theory and prior empirical work [10], predictors were organized into five domains that capture key aspects of adolescents’ developmental contexts. Within each domain, the specific variables were selected based on their theoretical relevance and availability in the dataset.

#### Sociodemographic predictors

Basic sociodemographic information was collected including age, sex (0 = *male*, 1 = *female*), parental highest educational level (1 = *illiterate or semi-literate*, 2 = *primary school*, 3 = *middle school*, 4 = *high school*, 5 = *2- or 3-year college*, 6 = *4-year college or bachelor’s degree*, 7 = *master’s degree or above*) and subjective socioeconomic status (SES). Participants’ subjective SES was assessed using the MacArthur Scale of Subjective Social Status [45]. The participants were instructed to evaluate their social status in comparison to others in the city and choose the rung that best matched their social standing. A higher score therefore indicates a higher level of subjective SES.

#### Extracurricular activity predictors

Participants were asked to estimate the number of hours they spent each week engaging in each of the activities, including physical activities, art, reading, and internet use.

#### Academic predictors

The mindset of intelligence scale was used to evaluate participants’ beliefs about intelligence [46], either as malleable or fixed. Secondly, participants’ educational aspiration was assessed by asking for the highest level of education that they hope to obtain, a widely used single-item measure in the literature [47]. In addition, six items were used to measure learning goals, including performance-oriented and mastery-oriented goals [48]. Finally, participants’ school engagement was measured by a 15-item scale assessing how they were behaviorally, cognitively, and emotionally engaged in activities at school [49].

#### Life experience predictors

Participants rated the extent to which they encountered a list of life stressors, including family, peer, and academic stress, which are the most relevant stressors among adolescents based on the literature [50]. Besides, they were also asked to evaluate and quantify positive early life experiences utilizing the 10-item Benevolent Childhood Experience Checklist [51].

#### Resilience predictors

**Fig. 1:**
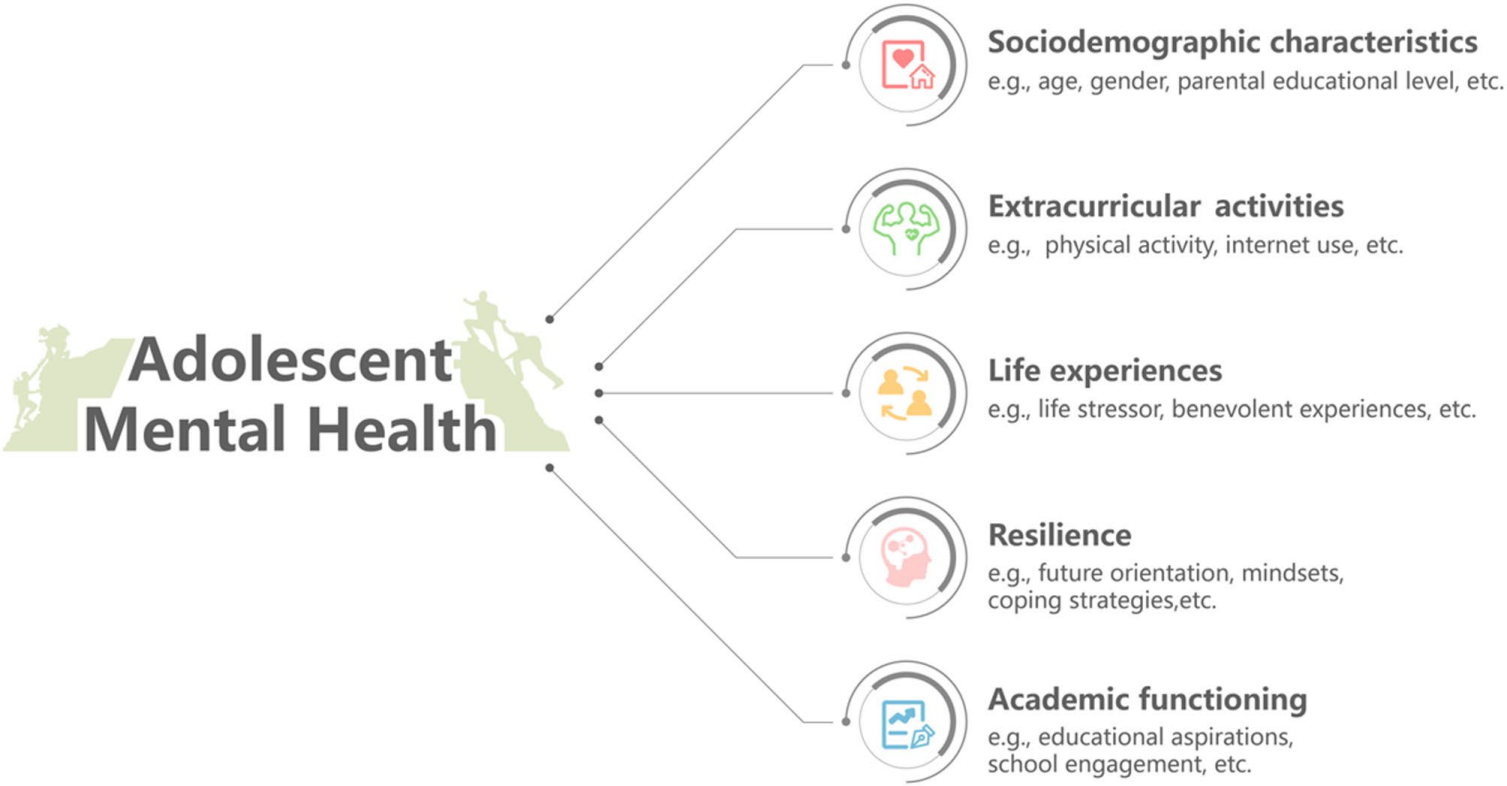
Conceptual framework of the current study

Participants reported their optimism towards the future and sense of meaning and purpose in life on short scales used in previous research and national surveys [52, 53]. In addition, participants’ gender norm attitude was measured by the subscale of the gender norm instrument, which mainly focused on gender stereotype traits [54]. Moreover, the three-item perceived income inequality scale was used to measure the perceived inequality at school [55]. Participants’ beliefs about stress, either as beneficial or debilitating, were measured using the 8-item Stress Mindset Measure-General (SMM-G) [56]. Additionally, the 12-item Highly Sensitive Child Scale was used to assess participants’ environmental sensitivity [57]. Participants also reported their emotion regulation skills, including two subscales of expressive suppression and cognitive reappraisal [58]. Finally, participants completed the 13-item shift-and-persist questionnaire to assess the frequency with which they adjusted to stressors or adversities (i.e., shift) and confronted adversity with perseverance (i.e., persist) [59]. Detailed psychometric information for all predictors is provided in the supplementary material. Figure 1 presents the conceptual framework of the current study.

### Statistical analysis

Bayesian Additive Regression Trees (BART), a state-of-the-art machine learning technique, was employed to determine and rank important predictors of adolescent mental health outcomes [36]. BART has demonstrated better predictive performance in numerous research areas when compared to other machine learning methods such as random forests and boosted models [31, 34]. BART is a Bayesian “sum-of-trees” model that uses regularization priors to constrain each tree. Specifically, the outcome is modeled as a sum of many regression trees, each of which makes a small contribution to the overall fit. Regularization priors constrain individual trees to remain shallow “weak learners,” which prevents overfitting while allowing the ensemble to flexibly capture complex, nonlinear, and interactive effects [31, 36, 60]. Inference is carried out through a Markov chain Monte Carlo (MCMC) algorithm that samples from the posterior distribution of the ensemble, providing reliable estimates and measures of uncertainty. In our analyses, outcomes are treated as continuous variables in the machine learning analyses. All predictors were used in their original units. For each outcome, we used 1250 posterior draws for each MCMC, eliminating the first 250 as burn-in to ensure a steady process. The default hyperparameters of the *bartMachine* package (50 trees, *k* = 2, *α* = 0.95, *β* = 2) were applied, consistent with the recommendations of Chipman et al. (2010) and prior research demonstrating their stability [36]. Cross-validation for the number of trees was not conducted, as earlier studies suggest that 50 trees typically provide an effective balance between predictive accuracy and computational efficiency [60].

To evaluate predictor importance, we used the framework developed by Bleich et al. (2014), which computes variable inclusion proportions (VIPs)—the proportion of times a predictor is selected as a splitting rule across posterior samples [31]. To reduce likelihood of spurious associations, null distributions of VIPs are generated through response permutation, providing principled thresholds for which predictors are truly important. Predictors were then ranked by their VIP values, with a higher value indicates higher importance for a specific predictor. Model performance was assessed using two complementary metrics: Pseudo-R², which represents the proportion of variance explained by the model, and Root Mean Squared Error (RMSE), which quantifies the average prediction error; both metrics have been widely used in previous research [34, 61]. Finally, marginal associations between the top predictors and the outcome were illustrated using the partial dependence functions, which show how each predictor relates to the outcome after accounting for all other predictors in the model. These plots estimate the expected outcome across the observed range of a given predictor partially out the contribution of other factors. This approach facilitates interpretation of BART results by illustrating both the direction and shape of predictor–outcome relationships [60]. All the analyses were performed using the *bartMachine* Package in R 4.3.1.

## Results

Of the 3,526 adolescents in the analytical sample, 42% reported clinically significant depressive symptoms, 56.5% reported mild or higher levels of anxiety, and 33.3% reported poor or unsatisfied sleep quality (see Table S2 for details). Moreover, depression and anxiety were positively correlated (*r* = 0.75, *p* < 0.001), while poor sleep quality was positively correlated with both anxiety (*r* = 0.42, *p* < 0.001) and depression (*r* = 0.53, *p* < 0.001).

**Fig. 2:**
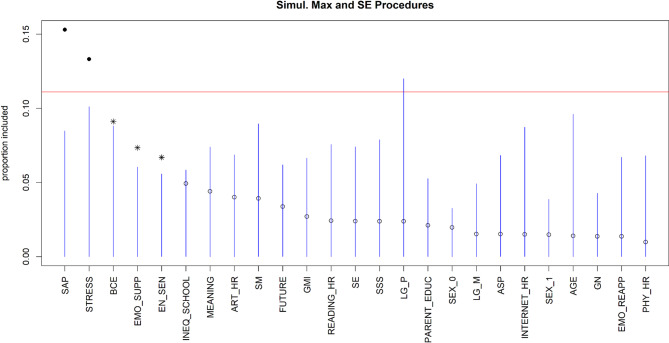
Visualization of the variable selection procedures for depressive symptoms. The standard threshold levels of the variable selection procedure are represented by the blue lines. The red line represents a more rigorous threshold, set at the 95th percentile of null importance values obtained from 10 permutations of the predictors. Solid dots represent variables that cross this threshold. Asterisks indicate variables that pass the blue line but do not reach the red line. An asterisk or a solid dot indicates the variables that have been selected, while unselected variables are indicated by open dots

Figure 2 presents the ranking of different factors that predict depressive symptoms, with the model showing good predictive performance (Pseudo-R² = 0.61, RMSE = 3.54). The top five predictors of depressive symptoms are the implementation of shift-and-persist strategies, experiences of life stressors and benevolent events, the use of expressive suppression strategies, as well as individual environmental sensitivity. The implementation of shift-and-persist strategies appeared to be the most important predictor as shown by the largest VIP value. We also explored marginal relationships between the top predictors and depressive symptoms (see Figure S1 in the supplemental material). The results suggested a generally linear relationship between shift-and-persist strategy, life stressors, benevolent experiences, environmental sensitivity, expressive suppression strategies and depressive symptoms.

**Fig. 3:**
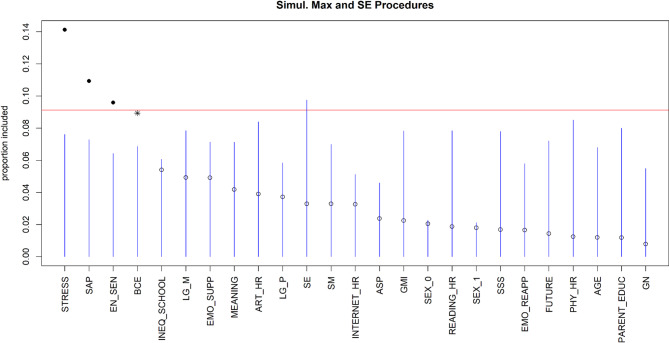
Visualization of the variable selection procedures for anxiety symptoms. The standard threshold levels of the variable selection procedure are represented by the blue lines. The red line represents a more rigorous threshold, set at the 95th percentile of null importance values obtained from 10 permutations of the predictors. Solid dots represent variables that cross this threshold. Asterisks indicate variables that pass the blue line but do not reach the red line. An asterisk or a solid dot indicates the variables that have been selected, while unselected variables are indicated by open dots

For anxiety symptoms (Pseudo-R² = 0.46, RMSE = 3.60), the top four predictors are experiences of life stressors, the implementation of shift-and-persist strategies, individual environmental sensitivity and benevolent experiences (see Fig. 3). Life stressor experiences appeared to be the most significant predictor, as indicated by the highest VIP value. The partial dependence plots of marginal effects on each variable with the highest importance on anxiety symptoms are presented in Figure S2, suggesting that individuals who experienced a greater number of life stressors, were more sensitive to environmental factors, had fewer benevolent experiences, and employed shift-and-persist strategies less frequently reported higher levels of anxiety symptoms.

**Fig. 4:**
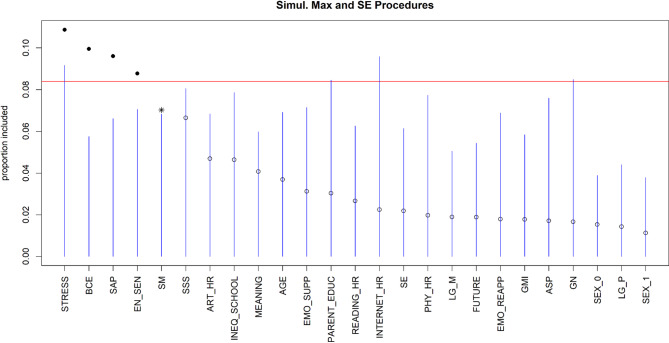
Visualization of the variable selection procedures for sleep quality. The standard threshold levels of the variable selection procedure are represented by the blue lines. The red line represents a more rigorous threshold, set at the 95th percentile of null importance values obtained from 10 permutations of the predictors. Solid dots represent variables that cross this threshold. Asterisks indicate variables that pass the blue line but do not reach the red line. An asterisk or a solid dot indicates the variables that have been selected, while unselected variables are indicated by open dots

For sleep quality (Fig. 4), the top five predictors are experiences of life stressors, benevolent events, the implementation of of shift-and-persist strategies, individual environmental sensitivity and stress mindset (Pseudo-R² = 0.24, RMSE = 0.65). As indicated by the highest VIP value, life stressor experiences again seem to be the most significant predictor. Further results suggested a linear relationship between shift-and-persist strategy, life stressors, benevolent experiences, environmental sensitivity, stress mindset and sleep quality (see Figure S3).

## Discussion

This study applies a Bayesian machine learning approach to identify and rank the protective and risk factors for various mental health outcomes in a sample of Chinese adolescents. Key risk factors included greater exposure to life stressors, higher environmental sensitivity, and greater reliance on expressive suppression strategies, whereas key protective factors included a stress-is-enhancing mindset, higher levels of benevolent experiences, and greater use of shift-and-persist coping strategies. Our findings are consistent with Bronfenbrenner’s ecological systems theory, which emphasizes that adolescent mental health is shaped by dynamic interactions across multiple levels of influence. The results highlight the salience of individual characteristics as well as microsystem factors within the family and school environment. By integrating both individual and contextual influences, our study underscores the importance of considering multi-level determinants when identifying risk and protective factors for adolescent mental health.

Key predictors varied across mental health indicators, but common factors—such as life stressors and benevolent experiences—emerged. These findings align with previous research on the impact of adversity [22, 62], and extend it by emphasizing the importance of benevolent experiences for sleep quality among adolescents. Furthermore, our findings demonstrate that positive experiences show robust associations with mental health, even when controlling for other factors. Future research could examine whether increasing benevolent experiences through school-based programs and parental guidance is associated with lower mental health risks.

Our results also showed that resilience-related characteristics—such as environmental sensitivity and shift-and-persist strategies—are strong common predictors of multiple outcomes. In contrast, expressive suppression and stress mindset are significant contributors to depressive symptoms and sleep quality of adolescents, respectively. These findings are consistent with earlier research highlighting the substantial effects of shift-and-persist strategies, emotion regulation, and environmental sensitivity on depression and anxiety [19, 20, 63]. Our results further suggest that shift-and-persist strategies may be associated with benefits beyond emotional well-being, extending to health-related behaviors such as sleep. Morever, while prior studies have demonstrated that individuals who are sensitive to the environment and those who have a stress-is-debilitating mindset are more likely to report higher depression or anxiety [20, 64]. The current findings added to the body of literature by highlighting the substantial associations of stress mindset and environmental sensitivity with sleep quality. Future research is warranted to explore the longitudinal association between environmental sensitivity, stress mindset and adolescents’ sleep quality. While our study primarily drew on an ecological systems framework to guide variable selection and interpretation, other theoretical perspectives may also provide complementary insights. For instance, emotion dysregulation frameworks may deepen understanding of the mechanisms through which regulation strategies such as expression suppression contribute to psychopathology [65]. Incorporating these perspectives in future work could enrich the interpretation of complex, multidomain predictors identified by the current study.

For adolescents, a sensitive period for mental health risks but also “windows of opportunity” for early intervention [66], these findings collectively highlight the potential value of psychological characteristics, especially those that are malleable, in directing screening practices and guiding future intervention strategies aimed at promoting mental health. Patterns such as heightened environmental sensitivity, limited use of shift-and-persist strategies, or a stress-is-debilitating mindset may serve as indicators of adolescents at greater risk and could benefit from closer monitoring and targeted support. Policy changes to enhance access to high-intensity treatments for mental health are still necessary for adolescents residing in middle- or low-income regions. Nevertheless, implementing these changes could be a protracted process. Future research could examine whether brief psychological interventions that promote resilience delivered through various youth care systems (e.g., primary care, and school programs) would supplement existing support systems and provide benefits for adolescents who currently lack access to additional care. Moreover, given that the outcomes assessed in this study are closely associated with suicidal behavior [67, 68], future research could explore whether the psychological characteristics identified are also pertinent to suicide prevention efforts at the educational, or community levels.

Several limitations should be noted when interpretating these findings. First, the cross-sectional design limits our ability to infer causality or bidirectional associations. As data collection in the Qu County Study continues, longitudinal data will allow us to test the predictive value of these factors for future mental health problems. Second, the sample is drawn from a single region in Southwestern China and is not nationally representative. Nonetheless, most participants came from middle- or lower-class backgrounds, providing valuable insight into the mental health challenges faced by socioeconomically disadvantaged adolescents in China. Future research should examine whether these findings generalize to populations in other cultural contexts and socioeconomic settings. Third, although BART is generally robust to multicollinearity and provides stable predictions, VIP and marginal associations should be interpreted with caution, as highly correlated predictors may conflate shared variance and obscure the unique contribution of individual variables. Finally, this study did not include all relevant variables or potential confounders, such as intelligence quotient, biological markers, or neurocognitive assessments. Incorporating objective measures—such as wearable devices to track daily activity and stress-related biomarkers—could enhance our understanding of predictors of adolescent mental health.

In conclusion, this study identified multidimensional predictors of various mental health outcomes in a large sample of Chinese adolescents. The findings indicate that life stressors, benevolent experiences, environmental sensitivity, shift-and-persist strategies, expressive suppression, and stress mindset were significantly associated with depressive symptoms, anxiety symptoms, and sleep quality among adolescents. Future research, particularly longitudinal and intervention studies, is needed to evaluate whether modifiable life experiences and resilience-related characteristics may help support adolescents’ mental well-being.

## Supplementary Information

Below is the link to the electronic supplementary material.

Supplementary Material 1.

## Data Availability

The de-identified dataset and analysis code are openly available on the Open Science Framework (https://osf.io/m4dhg/?view_only=6ec1064a6fd444f0a7b71f772d2ffd1d).

## References

[1] World Health Organization. Promoting mental health: concepts, emerging evidence, practice: summary report. Geneva: World Health Organization; 2004.

[2] GBD 2019 Mental Disorders Collaborators. Global, regional, and National burden of 12 mental disorders in 204 countries and territories, 1990–2019: a systematic analysis for the global burden of disease study 2019. Lancet Psychiatry. 2022;9:137–50. 35026139 10.1016/S2215-0366(21)00395-3 PMC8776563

[3] Kleinert S, Horton R. Adolescent health and wellbeing: a key to a sustainable future. Lancet. 2016;387:2355–6. 27174303 10.1016/S0140-6736(16)30297-5

[4] Racine N, McArthur BA, Cooke JE, et al. Global prevalence of depressive and anxiety symptoms in children and adolescents during COVID-19 A Meta-analysis. JAMA Pediatr. 2021;175:1142–50. 34369987 10.1001/jamapediatrics.2021.2482 PMC8353576

[5] Deng J, Zhou F, Hou W, et al. Prevalence of mental health symptoms in children and adolescents during the COVID-19 pandemic: a meta-analysis. Ann N Y Acad Sci. 2023;1520:53–73. 36537131 10.1111/nyas.14947 PMC9880764

[6] Sun H-L, Chen P, Zhang Q, et al. Prevalence and network analysis of internet addiction, depression and their associations with sleep quality among commercial airline pilots: a National survey in China. J Affect Disord. 2024;356:597–603. 38484881 10.1016/j.jad.2024.03.022

[7] Patalay P, Gage SH. Changes in millennial adolescent mental health and health-related behaviours over 10 years: a population cohort comparison study. Int J Epidemiol. 2019;48:1650–64. 30815691 10.1093/ije/dyz006 PMC6904321

[8] Shorey S, Ng ED, Wong CHJ. Global prevalence of depression and elevated depressive symptoms among adolescents: a systematic review and meta-analysis. Br J Clin Psychol. 2022;61:287–305. 34569066 10.1111/bjc.12333

[9] Orchard F, Gregory AM, Gradisar M, et al. Self-reported sleep patterns and quality amongst adolescents: cross-sectional and prospective associations with anxiety and depression. J Child Psychol Psychiatry. 2020;61:1126–37. 32557672 10.1111/jcpp.13288

[10] Bronfenbrenner U. Ecological systems theory. American Psychological Association; 2000.

[11] Courtney DB, Watson P, Krause KR et al. Predictors, moderators, and mediators associated with treatment outcome in randomized clinical trials among adolescents with depression a scoping review. JAMA Netw Open. 2022. 10.1001/jamanetworkopen.2021.46331 (Epub ahead of print 1 February 2022) 35103789 10.1001/jamanetworkopen.2021.46331 PMC8808324

[12] Lund HG, Reider BD, Whiting AB, et al. Sleep patterns and predictors of disturbed sleep in a large population of college students. J Adolesc Health. 2010;46:124–32. 20113918 10.1016/j.jadohealth.2009.06.016

[13] Magson NR, Freeman JYA, Rapee RM, et al. Risk and protective factors for prospective changes in adolescent mental health during the COVID-19 pandemic. J Youth Adolesc. 2021;50:44–57. 33108542 10.1007/s10964-020-01332-9 PMC7590912

[14] Marinova N, Rogers T, MacBeth A. Predictors of adolescent engagement and outcomes—a cross-sectional study using the togetherall (formerly big white Wall) digital mental health platform. J Affect Disord. 2022;311:284–93. 35588912 10.1016/j.jad.2022.05.058

[15] Wang D, Zhao J, Ross B, et al. Longitudinal trajectories of depression and anxiety among adolescents during COVID-19 lockdown in China. J Affect Disord. 2022;299:628–35. 34952127 10.1016/j.jad.2021.12.086 PMC8691948

[16] Wille N, Bettge S, Ravens-Sieberer U, et al. Risk and protective factors for children’s and adolescents’ mental health: results of the BELLA study. Eur Child Adolesc Psychiatry. 2008;17:133–47. 19132313 10.1007/s00787-008-1015-y

[17] Evans SE, Davies C, DiLillo D. Exposure to domestic violence: a meta-analysis of child and adolescent outcomes. Aggress Violent Behav. 2008;13:131–40.

[18] McLaughlin KA, Costello EJ, Leblanc W, et al. Socioeconomic status and adolescent mental disorders. Am J Public Health. 2012;102:1742–50. 22873479 10.2105/AJPH.2011.300477 PMC3482020

[19] Benner AD, Fernandez CC, Limon KL. Shifting and persisting in the face of life stressors: consequences for adolescent health. Appl Dev Sci. 2024;28:22–32. 38434488 10.1080/10888691.2022.2134131 PMC10904018

[20] Lionetti F, Klein DN, Pastore M, et al. The role of environmental sensitivity in the development of rumination and depressive symptoms in childhood: a longitudinal study. Eur Child Adolesc Psychiatry. 2022;31:1815–25. 34170421 10.1007/s00787-021-01830-6 PMC9666332

[21] Triana R, Keliat BA, Sulistiowati NMD. The relationship between self-esteem, family relationships and social support as the protective factors and adolescent mental health. Humanit Soc Sci Rev. 2019;7:41–7.

[22] Hou H, Zhang C, Tang J et al. Childhood experiences and psychological distress: can benevolent childhood experiences counteract the negative effects of adverse childhood experiences? Front Psychol.13. Epub ahead of print 25 February 2022. 10.3389/fpsyg.2022.800871 10.3389/fpsyg.2022.800871 PMC8914177 35282200

[23] Association AP. The road to resilience. Washington, DC: American Psychological Association;2014.

[24] Wiemken TL, Kelley RR. Machine learning in epidemiology and health outcomes research. Annu Rev Public Health. 2020;41:21–36. 31577910 10.1146/annurev-publhealth-040119-094437

[25] Chavanne AV, Paillère Martinot ML, Penttilä J, et al. Anxiety onset in adolescents: a machine-learning prediction. Mol Psychiatry. 2022;28:639–46. 36481929 10.1038/s41380-022-01840-z PMC9908534

[26] Garriga R, Mas J, Abraha S, et al. Machine learning model to predict mental health crises from electronic health records. Nat Med. 2022;28:1240–8. 35577964 10.1038/s41591-022-01811-5 PMC9205775

[27] O’Callaghan VS, Couvy-Duchesne B, Strike LT, et al. A meta-analysis of the relationship between subjective sleep and depressive symptoms in adolescence. Sleep Med. 2021;79:134–44. 33524839 10.1016/j.sleep.2021.01.011

[28] Gregory AM, Sadeh A. Annual research review: sleep problems in childhood psychiatric disorders—a review of the latest science. J Child Psychol Psychiatry. 2016;57:296–317. 26412255 10.1111/jcpp.12469

[29] Zhong Y, He J, Luo J, et al. A machine learning algorithm-based model for predicting the risk of non-suicidal self-injury among adolescents in Western china: a multicentre cross-sectional study. J Affect Disord. 2024;345:369–77. 37898476 10.1016/j.jad.2023.10.110

[30] Feghali R, El-Hachem C, Bakhos G, et al. The impact of economic crisis on the mental health of children and adolescents: a systematic review. Asian J Psychiatry. 2025;110:104613. 10.1016/j.ajp.2025.104613 40633184

[31] Bleich J, Kapelner A, George EI, et al. Variable selection for BART: an application to gene regulation. Ann Appl Stat. 2014;8:1750–81.

[32] Hu L, Gu C, Lopez M, et al. Estimation of causal effects of multiple treatments in observational studies with a binary outcome. Stat Methods Med Res. 2020;29:3218–34. 32450775 10.1177/0962280220921909 PMC7534201

[33] Hu L, Liu B, Li Y. Ranking sociodemographic, health behavior, prevention, and environmental factors in predicting neighborhood cardiovascular health: a bayesian machine learning approach. Prev Med. 141. Epub ahead of print December 2020. 10.1016/j.ypmed.2020.106240 10.1016/j.ypmed.2020.106240 PMC7704682 32860821

[34] Hu L, Liu B, Ji J et al. Tree-Based machine learning to identify and understand major determinants for stroke at the neighborhood level. J Am Heart Assoc. 9. Epub ahead of print 17 November 2020. 10.1161/JAHA.120.016745 10.1161/JAHA.120.016745 PMC7763737 33140687

[35] Niu L, Hu L, Li Y et al. Correlates of cancer prevalence across census tracts in the united states: A bayesian machine learning approach. Spat Spatio-Temporal Epidemiol. 42. Epub ahead of print August 2022. 10.1016/j.sste.2022.100522 10.1016/j.sste.2022.100522 PMC12906668 35934328

[36] Chipman HA, George EI, McCulloch RE. BART: Bayesian additive regression trees. Ann Appl Stat. 2010;4:266–98.

[37] Andresen EM, Malmgren JA, Carter WB, et al. Screening for depression in well older adults: evaluation of a short form of the CES-D. Am J Prev Med. 1994;10:77–84. 8037935

[38] Wang J, Wang T, Cheng Y. The development of depressive symptoms in subthreshold depression adolescents with a history of childhood maltreatment: a longitudinal network analysis. J Affect Disord. 2025;385:119389. 40350090 10.1016/j.jad.2025.119389

[39] Cheng HG, Chen S, McBride O, et al. Prospective relationship of depressive symptoms, drinking, and tobacco smoking among middle-aged and elderly community-dwelling adults: results from the China health and retirement longitudinal study (CHARLS). J Affect Disord. 2016;195:136–43. 26895091 10.1016/j.jad.2016.02.023

[40] Rutter LA, Brown TA. Psychometric properties of the generalized anxiety disorder Scale-7 (GAD-7) in outpatients with anxiety and mood disorders. J Psychopathol Behav Assess. 2017;39:140–6. 28260835 10.1007/s10862-016-9571-9 PMC5333929

[41] Löwe B, Decker O, Müller S et al. Validation and Standardization of the Generalized Anxiety Disorder Screener (GAD-7) in the General Population. Med Care. 46. Epub ahead of print 2008. 10.1097/MLR.0b013e318160d093 10.1097/MLR.0b013e318160d093 18388841

[42] Buysse DJ, Reynolds CF 3rd, Monk TH, et al. The Pittsburgh sleep quality index: a new instrument for psychiatric practice and research. Psychiatry Res. 1989;28:193–213. 2748771 10.1016/0165-1781(89)90047-4

[43] Chang L-Y, Wu C-C, Yen L-L, et al. The effects of family dysfunction trajectories during childhood and early adolescence on sleep quality during late adolescence: resilience as a mediator. Soc Sci Med. 2019;222:162–70. 30641286 10.1016/j.socscimed.2019.01.010

[44] Tsai P-S, Wang S-Y, Wang M-Y, et al. Psychometric evaluation of the Chinese version of the Pittsburgh sleep quality index (CPSQI) in primary insomnia and control subjects. Qual Life Res. 2005;14:1943–52. 16155782 10.1007/s11136-005-4346-x

[45] Goodman E, Adler N, Kawachi I et al. Adolescents’ perceptions of social status: Development and evaluation of a new indicator. Pediatrics. 108. Epub ahead of print August 2001. 10.1542/peds.108.2.e31 10.1542/peds.108.2.e31 11483841

[46] Dweck CS. Self-theories and goals: their role in motivation, personality, and development. Nebr Symp Motiv. 1990;38:199–235. 2130257

[47] Agger C, Meece J, Byun S. The influences of family and place on rural adolescents’ educational aspirations and Post-secondary enrollment. J Youth Adolesc. 2018;47:2554–68. 30062628 10.1007/s10964-018-0893-7

[48] Lau K-L, Lee JCK. Validation of a Chinese achievement goal orientation questionnaire. Br J Educ Psychol. 2008;78:331–53. 18039430 10.1348/014466507X238608

[49] Li Y, Agans JP, Chase PA, et al. School engagement and positive youth development: a relational developmental systems perspective. Teach Coll Rec. 2014;116:37–57.

[50] Xu J, Wang H, Liu S, et al. Relations among Family, Peer, and academic stress and adjustment in Chinese adolescents: a daily diary analysis. Dev Psychol. 2023;59:1346–58. 37199929 10.1037/dev0001538

[51] Narayan AJ, Rivera LM, Bernstein RE, et al. Positive childhood experiences predict less psychopathology and stress in pregnant women with childhood adversity: a pilot study of the benevolent childhood experiences (BCEs) scale. Child Abuse Negl. 2018;78:19–30. 28992958 10.1016/j.chiabu.2017.09.022

[52] Bryan A, Aiken L, West S. HIV/STD risk among incarcerated adolescents: optimism about the future and self-esteem as predictors of condom use self-efficacy. J Appl Soc Psychol. 2004;34:912–36.

[53] OECD. PISA 2018 Results (Volume III): What school life means for students’ lives. Epub ahead of print 2019. 10.1787/acd78851-en

[54] Moreau C, Li M, Ahmed S, et al. Assessing the spectrum of gender norms perceptions in early adolescence: a cross-cultural analysis of the global early adolescent study. J Adolesc Health. 2021;69:S16–22. 34217454 10.1016/j.jadohealth.2021.03.010

[55] Sommet N, Elliot AJ, Jamieson JP, et al. Income inequality, perceived competitiveness, and approach-avoidance motivation. J Pers. 2019;87:767–84. 30284720 10.1111/jopy.12432

[56] Crum AJ, Salovey P, Achor S. Rethinking stress: the role of mindsets in determining the stress response. J Pers Soc Psychol. 2013;104:716–33. 23437923 10.1037/a0031201

[57] Pluess M, Assary E, Lionetti F, et al. Environmental sensitivity in children: development of the highly sensitive child scale and identification of sensitivity groups. Dev Psychol. 2018;54:51–70. 28933890 10.1037/dev0000406

[58] Gross J, John O. Individual differences in two emotion regulation processes: implications for affect, relationships, and well-being. J Pers Soc Psychol. 2003;85:348–62. 12916575 10.1037/0022-3514.85.2.348

[59] Chen E, McLean KC, Miller GE. Shift-and-Persist strategies: associations with socioeconomic status and the regulation of inflammation among adolescents and their parents. Psychosom Med. 2015;77:371–82. 26167560 10.1097/psy.0000000000000157 PMC5890430

[60] Kapelner A, Bleich J. bartMachine: machine learning with bayesian additive regression trees. J Stat Softw. 2016;70:1–40.

[61] Zhang T, Geng G, Liu Y, et al. Application of bayesian additive regression trees for estimating daily concentrations of PM2.5 components. Atmosphere. 2020;11:1233. 34322279 10.3390/atmos11111233 PMC8315111

[62] Merrick JS. Risk and promotive factors for perinatal mental health problems: adverse and benevolent childhood experiences and contemporaneous support and stress;2023. 10.1017/S095457942400097X 39169778

[63] Mura F, Patron E, Messerotti Benvenuti S, et al. The influence of emotion regulation on the association between depression and heart rate variability in cardiac patients. Psychosom Med. 2022;84:702–10. 35412515 10.1097/PSY.0000000000001077

[64] Jiang Y, Zhang J, Ming H, et al. Stressful life events and well-being among rural-to-urban migrant adolescents: the moderating role of the stress mindset and differences between genders. J Adolesc. 2019;74:24–32. 31125950 10.1016/j.adolescence.2019.05.005

[65] Beauchaine TP, Cicchetti D. Emotion dysregulation and emerging psychopathology: a transdiagnostic, transdisciplinary perspective. Dev Psychopathol. 2019;31:799–804. 31290735 10.1017/S0954579419000671

[66] Uhlhaas PJ, Davey CG, Mehta UM, et al. Towards a youth mental health paradigm: a perspective and roadmap. Mol Psychiatry. 2023;28:3171–81. 37580524 10.1038/s41380-023-02202-z PMC10618105

[67] Busby Grant J, Batterham PJ, McCallum SM, et al. Specific anxiety and depression symptoms are risk factors for the onset of suicidal ideation and suicide attempts in youth. J Affect Disord. 2023;327:299–305. 36764362 10.1016/j.jad.2023.02.024

[68] Pirkis J, Bantjes J, Dandona R, et al. Addressing key risk factors for suicide at a societal level. Lancet Public Health. 2024;9:e816–24. 39265612 10.1016/S2468-2667(24)00158-0

