# Supplementary material for: Ranking factors across multiple domains in predicting adolescent mental health: a Bayesian machine learning approach
Source: Child Adolesc Psychiatry Ment Health. 2025 Oct 14;19:111. doi: 10.1186/s13034-025-00969-3 (PMC12519815; doi:10.1186/s13034-025-00969-3)
Supplement: Supplementary file 1 — Supplementary Material 1. [file 13034_2025_969_MOESM1_ESM.docx]

**Ranking Factors across Multiple Domains in Predicting Adolescent Mental Health: A Bayesian Machine Learning Approach**

**Measures for the predictors**

***Academic Functioning***

***Mindset of intelligence.*** The mindset of intelligence scale was used to evaluate participants’ beliefs about intelligence [1], and is a widely used measure in both adolescent and Chinese populations [2]. The scale consists of four entity statements (e.g., “Intelligence is something that cannot be changed very much”). Participants were asked to respond on a 6-point Likert scale ranging from 1 (*strongly disagree*) to 6 (*strongly agree*). The items were reverse-coded first, and a higher mean score reflects a stronger growth mindset in intelligence. The scale demonstrated satisfactory internal consistency, with Cronbach’s α = 0.74 and McDonald’s ω = 0.74.

***Educational aspiration.*** Participants’ educational aspiration was measured with one item: “What is the highest level of education you hope that you will attain?”. Respondents were given a 6-point Likert scale (1 = middle school, 2 = high school, 3 = 2- or 3-year college, 4 = 4-year college or bachelor's degree, 5 = master's degree, and 6 = doctoral degree). Higher scores on this item, therefore, indicate an aspiration for a higher level of education. Based on previous nationally and large-scale research, using a single item has been considered reliable for assessing participants' educational aspirations [3, 4].

***Learning goals.*** Six items from previous research were used to assess participants’ learning goals [5], including mastery-oriented goals and performance oriented goals. This scale has been successfully used in previous studies to measure constructs among Chinese adolescents [6]. Mastery-oriented goals were measured by three statements (e.g., “I want to do better than other students in my class.”) and performance oriented goals were assessed with three statements (e.g., “I want to do better than other students in my class”). Respondents were given a 6-point Likert scale, ranging from 1 (strongly disagree) to 6 (strongly agree). A higher mean score indicates a higher mastery-oriented goal or performance-oriented goal, respectively. The scale showed satisfactory internal consistency in this study, with mastery-oriented goals demonstrating Cronbach’s α = 0.82 and McDonald’s ω = 0.82, and performance-oriented goals demonstrating Cronbach’s α = 0.76 and McDonald’s ω = 0.77.

***School engagement.*** Participants’ school engagement was assessed using the 15-item scale, which assessed three aspects of engagement at school: emotional (e.g., “Feeling part of my school”), behavioral (e.g., “Completing homework on time”), and cognitive (e.g., “The things I learn at school are useful) engagement [7]. Participants were asked to rate how frequently they agreed with each statement, on a 4-point Likert scale, ranging from 1 (*never*) to 4 (*always*). The scale was validated in the Chinese population [8]. The scale demonstrated good overall reliability, with a Cronbach's alpha (α) of 0.76 and McDonald's omega (ω) of 0.77.

***Life Experiences***

***Life stressors.*** Participants rated to what extent they experienced family, peer, and academic stress on a 4-point Likert scale, ranging from 1 (definitely false) to 4 (definitely true) [9]. This scale has demonstrated good construct validity and has been widely used in research on adolescents, and within Chinese populations [10]. Family stress was assessed by five items (e.g., “was punished or disciplined by parents”), peer stress was measured by four items (e.g., “was teased by another schoolmate"), and academic stress was measured by three items (e.g., “did not understand something taught in class”). A higher mean score indicates more life stressors the participant experiences in total. This scale demonstrated good overall reliability, with a Cronbach's alpha (α) of 0.81 and McDonald's omega (ω) of 0.82.

***Benevolent experiences.*** Positive early life experiences were evaluated and quantified utilizing the 10-item Benevolent Childhood Experience Checklist [11]. The responses to the listed statements (e.g., "Did you have at least one caregiver with whom you felt safe?") were binary-scored, with 0 indicating no experience and 1 indicating yes. Items were summed so that a higher total score indicates more benevolent experiences in the past.

***Resilience***

***Future orientation.*** We used the Chinese version of four-item scale assess participants’ future orientation, which has been shown to have good reliability and validity among Chinese adolescents [12]. Individuals were asked to rate the likelihood that each statement would be achieved in the future (e.g., “Get a good job someday”) on a 4-point Likert scale, ranging from 1 (*very unlikely*) to 4 (*very likely*). A higher mean score suggests a higher degree of optimism regarding the future. Cronbach's alpha and McDonald’s omega coefficients were 0.85.

***Meaning in life***. Three items were used to measure participants’ sense of meaning and purpose, which were developed and widely used in national sample surveys such as the OECD [13]. Participants rated how much they agreed with each statement on a 7-point Likert scale, ranging from 1 (strongly disagree) to 7 (strongly agree). Higher Mean scores indicate greater levels of meaning in life. Cronbach's alpha and McDonald’s omega coefficients were 0.82.

***Gender norm attitude.*** Participants’ perceptions of gender norms were measured using seven items assessed on a 5-point Likert scale, ranging from 1 (strongly disagree) to 5 (strongly agree) [14]. This scale has demonstrated good reliability and validity in Chinese adolescent populations [15]. Example items include “girls need their parents protection more than boys”. A higher mean score therefore reflects more stereotypical attitudes related to masculinities and femininities. This scale demonstrated good internal consistency, with Cronbach’s α = 0.84 and McDonald’s ω = 0.84.

***Perceived inequality.*** The three-item perceived income inequality scale, which has been well-validated in previous research, was adapted for this study (e.g., “In my school, there is a huge gap between rich and poor”) [16]. Respondents were given a 5-point Likert scale, ranging from 1 (strongly disagree) to 5 (strongly agree). A higher mean score suggests a greater degree of perceived inequality at school. The Inequality scale demonstrated satisfactory internal consistency, with Cronbach’s α = 0.82 and McDonald’s ω = 0.83.

***Stress mindset.*** Participants’ beliefs about stress were measured using the Chinese version 8-item Stress Mindset Measure-General (SMM-G) [17], which has been validated among Chinese university students [2]. The participants rated how strongly they agreed with each of the items, with four items measuring a stress-is-debilitating mindset and four items measuring a stress-is-enhancing mindset (e.g., “Experiencing stress improves my health and vitality”). Respondents were given a 5-point Lik debilitating ert scale, ranging from 0 (strongly disagree) to 4 (strongly agree). The stress-is- statements were reverse coded, and a higher mean score indicates a stronger stress-is-enhancing mindset. This scale demonstrated good internal consistency, with Cronbach’s α = 0.76 and McDonald’s ω = 0.76.

***Environmental sensitivity.*** The 12-item Highly Sensitive Child Scale was used to assess participants’ environmental sensitivity [18], which demonstrated good construct validity and has been widely applied in adolescent research [19]. It includes a range of reactions to environmental cues, for instance, finding it discomfort in the presence of a busy environment, enjoying pleasant smells and music, and being prone to unpleasant feelings caused by strong sensory stimuli (e.g., “Loud noises make me uncomfortable”). Respondents were given a 7-point Likert scale, ranging from 1 (*strongly disagree*) to 7 (*strongly agree*), with a higher average score representing higher levels of sensitivity towards the environment. This scale demonstrated acceptable internal consistency in the present study (Cronbach’s α = 0.72, McDonald’s ω = 0.72).

***Shift-and-Persist coping strategies.*** Participants completed the 13-item Shift-and-Persist Questionnaire to assess the frequency with which they adapted to stressors or adversities (i.e., shift) and confronted adversity with perseverance (i.e., persist) [20, 21]. Respondents were given a 4-point Likert scale, ranging from 1 (*not at all*) to 7 (*a lot*). Example items include “When something doesn't turn out the way I want, I try and find other ways to get to the goal that I had wanted”, with a higher average score representing greater implementation of shift-and-persist strategies. This scale has demonstrated good reliability and validity in prior research [22], and showed satisfactory internal consistency in the present study (Cronbach’s α = 0.87, McDonald’s ω = 0.87).

***Emotion regulation strategies.*** The 10-item Emotion Regulation Questionnaire for Children and Adolescents (ERQ-CA) was used, a measure that has demonstrated good reliability and validity in previous research [23, 24]. It consists of two dimensions: four items measure expressive suppression (e.g., “I keep my emotions to myself”) and six items measure cognitive reappraisal (e.g., “When I’m faced with a stressful situation, I make myself think about it in a way that helps me stay calm”). The responses were graded on a 7-point Likert scale ranging from 1 (*strongly disagree*) to 7 (*strongly agree*), with a higher average score indicating a higher likelihood of utilizing a particular emotion regulation strategy. This scale showed adequate internal consistency in the current study (cognitive reappraisal: Cronbach’s α = 0.76, McDonald’s ω = 0.78; expressive suppression: Cronbach’s α = 0.63, McDonald’s ω = 0.65).

**Table S1.** Descriptive information of the variables for Bayesian Additive Regression Trees (BART)

| **Domain** | **Variable Name** | **Definition** |
| --- | --- | --- |
| Sociodemographic  characteristics | SEX | %Male |
|  |  | %Female |
|  | AGE | Age of the participants |
|  | PARENT_EDUC | Parental highest educational level:  Middle school or below |
|  |  | High school |
|  |  | 2- or 3-year college |
|  |  | 4-year college or bachelor's degree or above |
|  | SSS | Participants’ subjective socioeconomic status |
| Extracurricular activities | PHY_HR | the number of hours participants spent each week engaging in sports |
|  | READING_HR | the number of hours participants spent each week engaging in reading |
|  | INTERNET_HR | the number of hours participants spent each week engaging in internet use |
|  | ART_HR | the number of hours participants spent each week engaging in art activity |
| Academic functioning | GMI | Mindset of intelligence |
|  | ASP | Educational aspiration:  High school or below |
|  |  | 2- or 3-year college |
|  |  | 4-year college or bachelor's degree |
|  |  | master’s degree or above |
|  | LG_P | Performance-oriented learning goal |
|  | LG_M | Mastery-oriented learning goal |
|  | SE | School engagement |
| Life experiences | STRESS | Experience of life stressors |
|  | BCE | Experience of benevolent events |
| Resilience | FUTURE | Future orientation |
|  | MEANING | Meaning in life |
|  | GN | Gender norm attitude |
|  | INEQ_SCHOOL | Perceived economic inequality |
|  | EN_SEN | Environmental sensitivity |
|  | SAP | Shift-and-persist strategies |
|  | EMO_SUPP | Emotion regulation strategies:  Expressive suppression |
|  | EMO_REAPP | Emotion regulation strategies: Cognitive reappraisal |
|  | SM | Stress mindset |

**Table S2.** Descriptive statistics of the mental health outcome variables

| **Domain** | **Variable** | **M (*SD*) / n (%)** | **Min-Max** |
| --- | --- | --- | --- |
| Mental health outcomes | Depressive symptoms |  |  |
|  | Below clinical level | 2,044 (58.0%) | 0-10 |
|  | Clinical level and above | 1,482 (42.0%) | 11-30 |
|  | Anxiety symptoms |  |  |
|  | No | 1,535 (43.5%) | 0-4 |
|  | Mild | 1,222 (34.7%) | 5-9 |
|  | Moderate | 532 (15.1%) | 10-14 |
|  | Severe | 237 (6.7%) | 15-21 |
|  | Sleep quality |  |  |
|  | Very good | 484 (13.7%) | - |
|  | Fairly good | 1868 (53.0%) |  |
|  | Fairly bad | 999 (28.3%) |  |
|  | Very bad | 175 (5%) |  |

**Fig S1.** Partial dependence plots for the top five predictors for depressive symptoms.


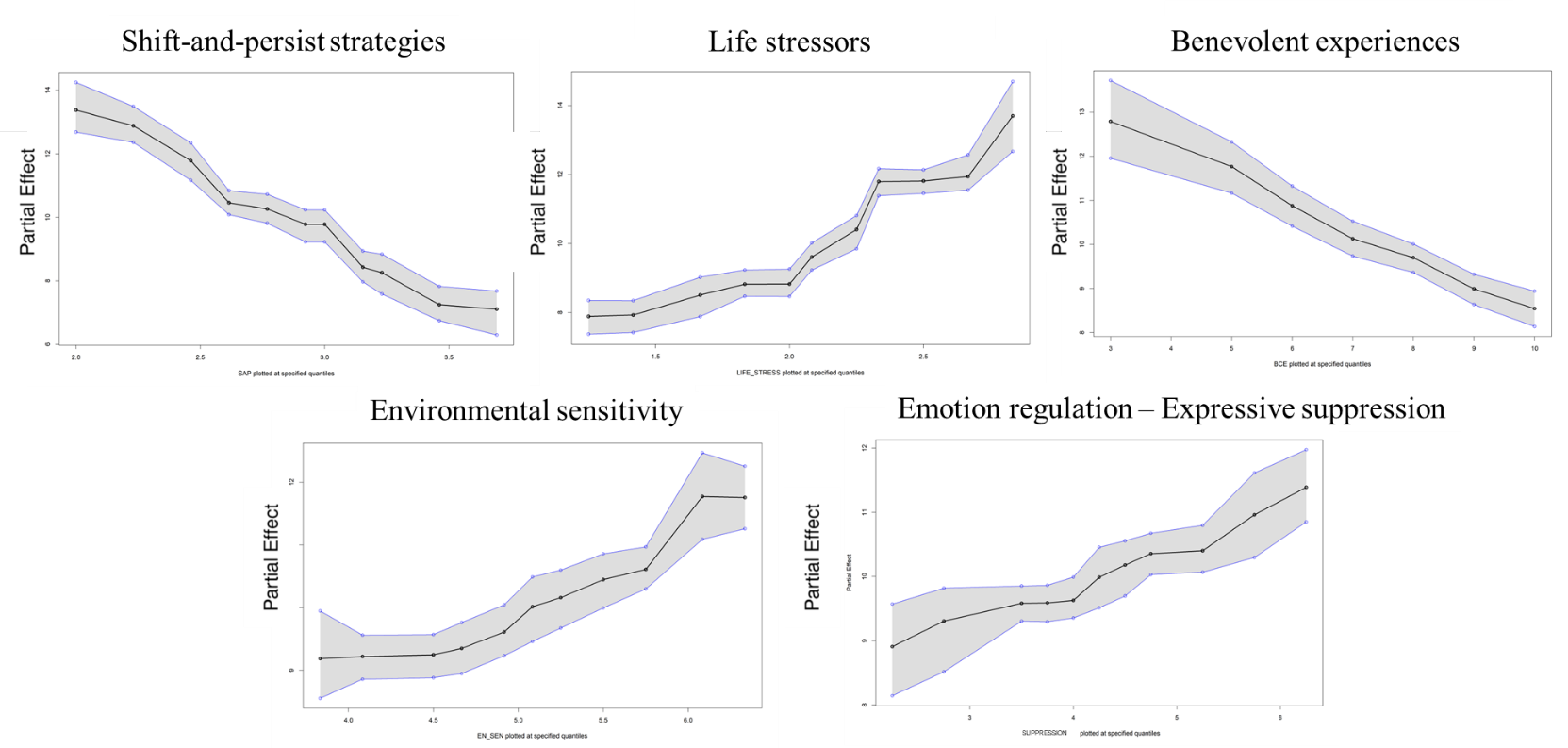


**Fig S2.** Partial dependence plots for the top four predictors for anxiety symptoms


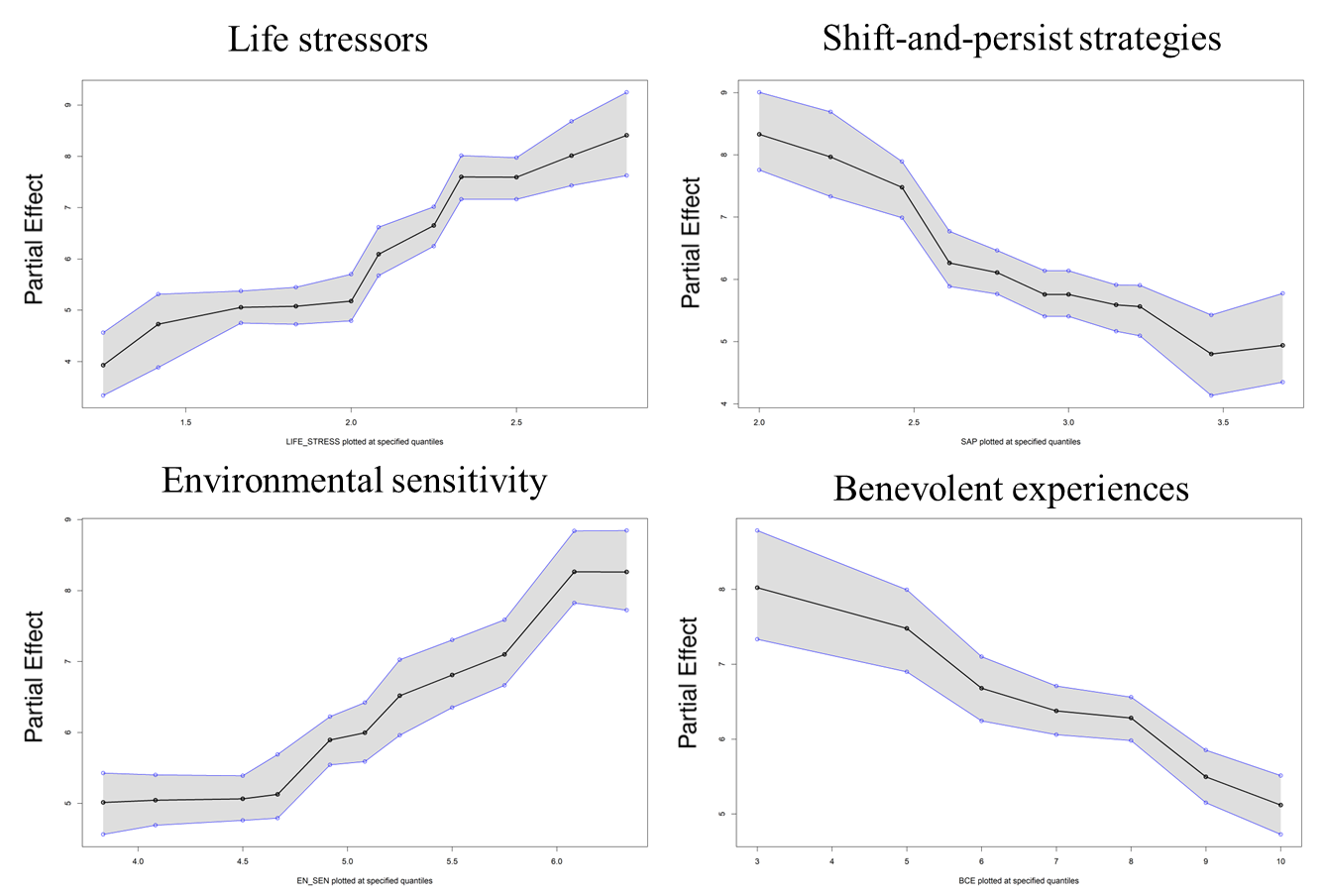


**
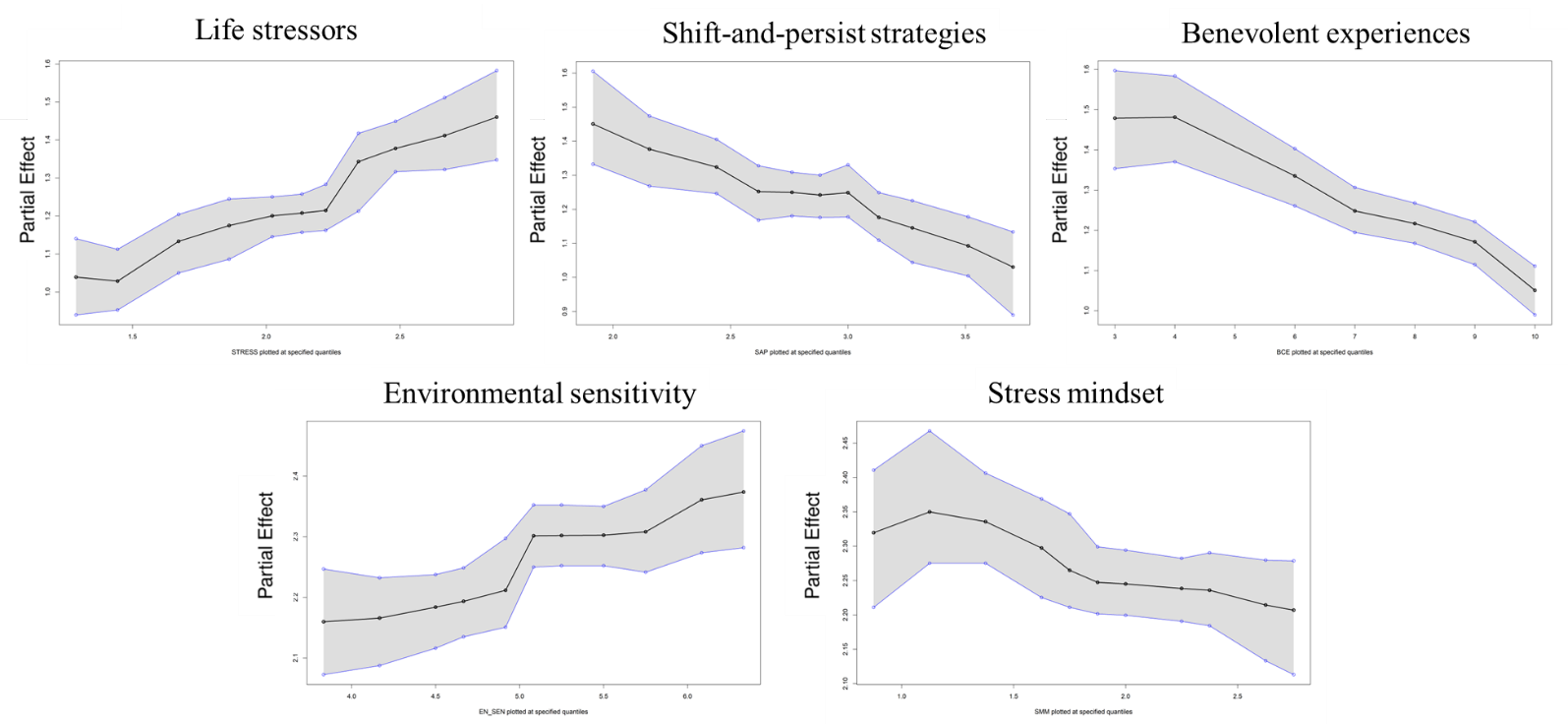
Fig S3.** Partial dependence plots for the top five predictors for poor sleep quality

**References**

[1] Dweck CS. Self-theories and goals: their role in motivation, personality, and development. *Nebr Symp Motiv* 1990; 38: 199–235.

[2] Zhao S, Zhang Y, Yu C, et al. Trajectories of Perceived Stress among Students in Transition to College: Mindset Antecedents and Adjustment Outcomes. *J Youth Adolesc* 2023; 52: 1873–1886.

[3] Agger C, Meece J, Byun S. The Influences of Family and Place on Rural Adolescents’ Educational Aspirations and Post-secondary Enrollment. *J Youth Adolesc* 2018; 47: 2554–2568.

[4] Garg R, Melanson S, Levin E. Educational aspirations of male and female adolescents from single-parent and two biological parent families: A comparison of influential factors. *J Youth Adolesc* 2007; 36: 1010–1023.

[5] Lau K-L, Lee JCK. Validation of a Chinese achievement goal orientation questionnaire. *Br J Educ Psychol* 2008; 78: 331–353.

[6] Zhang L, Mendoza NB, Jiang Y. From classroom goal structures to academic outcomes: The mediating role of expectancy-value beliefs in adolescent learning. *Eur J Psychol Educ* 2025; 40: 59.

[7] Li Y, Agans JP, Chase PA, et al. School engagement and positive youth development: A relational developmental systems perspective. *Teach Coll Rec* 2014; 116: 37–57.

[8] Ye Z, Wu K, Niu L, et al. Peer Victimization and School Engagement among Chinese Adolescents: Does Classroom-Level Victimization Matter? *J Youth Adolesc* 2025; 54: 1476–1488.

[9] Bai S, Repetti RL. Negative and Positive Emotion Responses to Daily School Problems: Links to Internalizing and Externalizing Symptoms. *J Abnorm Child Psychol* 2017; 46: 423–435.

[10] Xu J, Wang H, Liu S, et al. Relations Among Family, Peer, and Academic Stress and Adjustment in Chinese Adolescents: A Daily Diary Analysis. *Dev Psychol* 2023; 59: 1346–1358.

[11] Narayan AJ, Rivera LM, Bernstein RE, et al. Positive childhood experiences predict less psychopathology and stress in pregnant women with childhood adversity: A pilot study of the benevolent childhood experiences (BCEs) scale. *Child Abuse Negl* 2018; 78: 19–30.

[12] Bryan A, Aiken L, West S. HIV/STD risk among incarcerated adolescents: Optimism about the future and self-esteem as predictors of condom use self-efficacy. *J Appl Soc Psychol* 2004; 34: 912–936.

[13] OECD. PISA 2018 Results (Volume III): What school life means for students’ lives. Epub ahead of print 2019. DOI: 10.1787/acd78851-en.

[14] Moreau C, Li M, Ahmed S, et al. Assessing the Spectrum of Gender Norms Perceptions in Early Adolescence: A Cross-Cultural Analysis of the Global Early Adolescent Study. *J Adolesc Health* 2021; 69: S16–S22.

[15] Yu C, Zuo X, Lian Q, et al. Comparing the Perceptions of Gender Norms among Adolescents with Different Sibling Contexts in Shanghai, China. *Children* 2022; 9: 1281.

[16] Sommet N, Elliot AJ, Jamieson JP, et al. Income inequality, perceived competitiveness, and approach-avoidance motivation. *J Pers* 2019; 87: 767–784.

[17] Crum AJ, Salovey P, Achor S. Rethinking Stress: The Role of Mindsets in Determining the Stress Response. *J Pers Soc Psychol* 2013; 104: 716–733.

[18] Pluess M, Assary E, Lionetti F, et al. Environmental Sensitivity in Children: Development of the Highly Sensitive Child Scale and Identification of Sensitivity Groups. *Dev Psychol* 2018; 54: 51–70.

[19] Cao J, Xu X, Liu X, et al. Profiles of Family and School Experiences and Adjustment of Adolescents During the Transition to High School. *J Youth Adolesc* 2024; 53: 2002–2015.

[20] Chen E, McLean KC, Miller GE. Shift-and-Persist Strategies: Associations With Socioeconomic Status and the Regulation of Inflammation Among Adolescents and Their Parents. *Psychosom Med* 2015; 77: 371–382.

[21] Lam PH, Miller GE, Chiang JJ, et al. One size does not fit all: Links between shift-and-persist and asthma in youth are moderated by perceived social status and experience of unfair treatment. *Dev Psychopathol* 2018; 30: 1699–1714.

[22] Santo CD, Desmarais A, Christophe NK. Coping with ethnic-racial discrimination: Protective-reactive effects of shift-and-persist coping on internalizing symptoms among Black American adolescents. *J Res Adolesc* 2024; 34: 1420–1430.

[23] Gross J, John O. Individual differences in two emotion regulation processes: Implications for affect, relationships, and well-being. *J Pers Soc Psychol* 2003; 85: 348–362.

[24] Gong J, Wang M-C, Zhang X, et al. The Emotion Regulation Questionnaire for Children and Adolescents (ERQ-CA): Factor Structure and Measurement Invariance in a Chinese Student Samples. *J Pers Assess* 2022; 104: 774–783.
